# Employees and the National Insurance Act

**Published:** 1912-04-20

**Authors:** 


### NATIONAL INSURANCE ACT DEPARTMENT.

### EMPLOYEES AND THE NATIONAL INSURANCE ACT.

### VII.—Benefits Receivable under the Act—(*continued*).

THE ADDITIONAL BENEFITS.

We have already dealt briefly with the first seven clauses in the list of additional benefits allowed by the Act, as set out in the Fourth Schedule, Part II. The general character of these first seven benefits is that of an extension of the standard benefits and an increase in the rates at which they are payable. For this reason it is unlikely that any society will include any one of these particular benefits in an initial scheme as a substitute for sickness or disablement benefits. They will only become operative as a means of distributing surplus when this is shown to exist upon an actuarial valuation.

The next item in the list does not allow of any choice on the part of an individual member.

The clause in question allows the building or leasing of premises suitable for convalescent homes, and the maintenance of such homes. With the improved medical treatment and attendance foreshadowed by the Act there will doubtless come an increased appreciation of the work of convalescent homes, and the necessity will probably arise of adding to their number. Not only will the individual member who is sent to such a home be benefited thereby, but the society as a whole may obtain an advantage in the diminished charge for sickness and disablement benefits that will accrue by the complete restoration to health of its sick members. It would seem that the clause can only apply to societies with large numbers of members, as it is unlikely that any but the largest societies will have sufficient funds to build or lease a home of their own. The societies are, however, empowered by the Act, quite apart from the scheme of additional benefits, to grant such subscriptions or donations as they may think fit to hospitals, dispensaries, and other charitable institutions, or for the support of district nurses. Subscriptions and donations of this character usually carry the privilege of nomination of members for treatment in the particular institution, and district nurses supported by a society would, of course, be at the service of members when required. In this way indirect benefit is obtained by the society as a whole, and it is to be hoped that this fact will not be lost sight of by the approved societies when they come into full operation. Although the provision of convalescent homes can hardly be classed as an extension of the standard benefits, it is so closely akin to the sickness and disablement allowances that it cannot be said to meet the case of a compulsory contributor, for whom adequate provision in case of sickness is already made.

The first satisfactory alternative to sickness and disablement benefits is allowed by the next clause, numbered (9), which makes it possible for an approved society to offer a scheme for the payment of pensions or superannuation allowances, either by way of addition to old-age pensions under the Old Age Pensions Act, or otherwise. A large society may find it possible to undertake the granting of such pensions and to make the payments itself, but it is probable that only societies with large numbers of members taking this form of benefit will be able to do so, because the liability, being of a deferred nature, will depend upon actuarial estimates, and these require a sufficient number of members to form an average in order that they may eventually be realised. In order to meet the case of smaller societies, clause 10 allows the additional benefit to be applied, subject to prescribed conditions, in payment of contributions to superannuation funds in which the members are interested. When the contributions are paid in this way the society will itself be released from the liability for the payment of the future pension, and the liability will rest on the superannuation fund to which the contributions are paid. It will be remembered that an approved society must not be carried on for profit to shareholders or promoters, and as this is one of the fundamental conditions to be fulfilled before approval is granted, it would seem that any superannuation fund to which the contributions, in substitution for sickness and disablement benefits, may be applied will also have to be of the character of a mutual benefit society.

There are several very important points that should be carefully considered by each individual member of an approved society before deciding whether to take the standard sickness and disablement benefits or the alternative superannuation allowance offered by the society. The first of these matters is the permanency of the present employment or class of employment, and whether the provision of treatment and maintenance during sickness at present granted by the employer will be continued indefinitely in future.

If the conditions of employment are such that maintenance and treatment during incapacity are guaranteed, either explictly or by implication, there would seem to be no immediate need of the insurance against sickness; but it must not be forgotten that circumstances may necessitate a change of occupation, and no such provision during sickness may be allowed in the new employment. Although wages may be continued during illnesses of short duration, it is only in very few classes of employment where the employer would continue the payments in cases of prolonged incapacity. This is a most important matter for consideration, for while it may doubtless be possible for the societies to allow their members, when in good health to alter the form of benefit from the superannuation allowance back to the standard sickness and disablement benefits, it is clear that they could not allow such alteration in the case of a member with a bad sickness record, or for one whose incapacity for work had become chronic. It is in circumstances such as these that the standard disablement benefit is of value, for though the amount of 5s. per week may be small, it is very much better than nothing at all. Another matter to be carefully noted is that pensions and superannuation allowances are expensive benefits to provide, and that, as the contributions to sickness and disablement benefits are small, the alternative deferred payments cannot be large. The amounts of pension must necessarily be graded according to age, both in regard to the age at which the insurance is taken up, and also with reference to the age at which the pension is to become payable. The older the age at which the superannuation allowance is to commence the larger will be the amount of the benefit obtainable, not only because the contributions will have been paid for a longer period, but also because the number of survivors amongst whom the fund is to be distributed in the form of pensions will be reduced by deaths occurring during the interval.

Looking at the matter in another way, we may suppose, for the purpose of illustration, that the age at which the pension is to commence is fixed at seventy: the amount of the pension that could be allowed to a member joining at age forty would be very much less than that applicable to a person joining at age twenty.

As the pensions will most probably be granted on a mutual benefit plan, it may be necessary in the future for the estimated amounts to be revised. This will be the case if the rate of mortality actually experienced does not coincide with that expected. If fewer die than the number anticipated, the amounts of pension will be reduced; while if the actual mortality is greater, the benefit should be increased.

### Where our Readers can be Helped.

All these considerations should be borne in mind when the time comes for the choice of benefits, and it is in such matters as this that independent advice, through the medium of our columns, is likely to be of service to our readers. If they will furnish us with particulars of any scheme that is put before them, and indicate the points on which advice is desired, we shall be pleased to do whatever we can in order to assist in obtaining the most suitable form of benefit. The coupon which will be found on the back cover should be sent with the letter of inquiry.

Section 11 of the additional benefits schedule allows payments to members who are in want or distress, including the remission of arrears whenever such arrears may have become due. In adopting such a plan for the disposal of surplus, a society would be bound to impose stringent restrictions as to its application. The circumstances of each individual application for help would require careful scrutiny in order to preclude the possibility of improper use of the funds at disposal. The benefit is of a general character and would have to be adopted by a society as a whole; it could not be allowed as an alternative to the standard sickness and disablement allowances, at the option of an individual member.

When a member of an approved society is ill and is an inmate of a voluntary hospital or similar institution, the standard sickness or disablement allowance will not be payable for his personal use. If he has dependants, the Act provides that the sum which would have been payable except for the fact that he is an inmate of such institution, shall be applied in whole or in part towards the relief or maintenance of the dependants. If he has no dependants, the society may, by agreement with the institution, pay the amount of the benefit to the institution towards the cost of his maintenance. Under the additional benefits schedule it will also be possible for payments to be made for the personal use of a member who, by reason of being an inmate of a hospital or other institution, is not in receipt of sickness or disablement benefit.

The standard sickness benefit is only payable when the insured person is rendered incapable of work by some specific disease or by bodily or mental disablement. There are circumstances, however, in which the member is not himself incapable of work, but by reason of infection at home he is debarred from attendance at his usual employment and in consequence suffers loss of wages. No payment can be made from the standard benefits in such a case, but the Act empowers a society to include amongst its additional benefits the provision of payments to members in the circumstances mentioned. This is another form of benefit which it would seem must apply to all members, and not to individuals at their option.

The last item in the list allows as an additional benefit the repayment of the whole or any part of the contributions thereafter payable under the Act by members of the society, or any special class of members. This would seem to indicate the possibility of the contributions ceasing, so far as the member is concerned, at an earlier age than seventy. In the normal case of an employed contributor the contributions are payable until the attainment of age seventy. No contributions become due whilst the member is actually in receipt of sickness or disablement benefit, but they continue to become due though the member may be unemployed, so long as that unemployment is not caused by incapacity for work arising through disease or disablement. Most persons are unable to sustain the full stress of continuous occupation until age seventy, and the prospect of limiting the payments to cease at, say, age sixty-five, which may later on be attained, is an attractive one. Our readers will be well advised to see that the society they choose is thoroughly alive to the advantages of this form of additional benefit.

---

NOTE :—Questions and Correspondence on all doubtful points are invited, and should be addressed to the Insurance Editor, The Hospital, 28 Southampton Street, Strand, W.C.

### QUESTIONS AND ANSWERS.

*Answers to Questions on Insurance matters will be found on page* 69.

